# Correction: Nickel–cobalt hydroxide: a positive electrode for supercapacitor applications

**DOI:** 10.1039/d5ra90065d

**Published:** 2025-05-21

**Authors:** M. Sangeetha Vidhya, G. Ravi, R. Yuvakkumar, Dhayalan Velauthapillai, M. Thambidurai, Cuong Dang, B. Saravanakumar

**Affiliations:** a Nanomaterials Laboratory, Department of Physics, Alagappa University Karaikudi Tamil Nadu 630 003 India yuvakkumarr@alagappauniversity.ac.in; b Faculty of Engineering and Science, Western Norway University of Applied Sciences Bergen – 5063 Norway; c Centre for OptoElectronics and Biophotonics (COEB), School of Electrical and Electronic Engineering, The Photonics Institute (TPI), Nanyang Technological University 50 Nanyang Avenue Singapore; d School for Advanced Research in Polymers (SARP), Laboratory for Advanced Research in Polymeric Materials (LARPM), Central Institute of Plastics Engineering & Technology (CIPET) Bhubaneswar – 751024 India

## Abstract

Correction for ‘Nickel–cobalt hydroxide: a positive electrode for supercapacitor applications’ by M. Sangeetha Vidhya *et al.*, *RSC Adv.*, 2020, **10**, 19410–19418, https://doi.org/10.1039/D0RA01890B.

The authors regret an error in Fig. 3 of the original article where the data for Co-Ni(OH)_2_ was accidentally used for Ni(OH)_2_. The corrected Fig. 3 is shown below with the original data for Ni(OH)_2_.

**Fig. 3:**
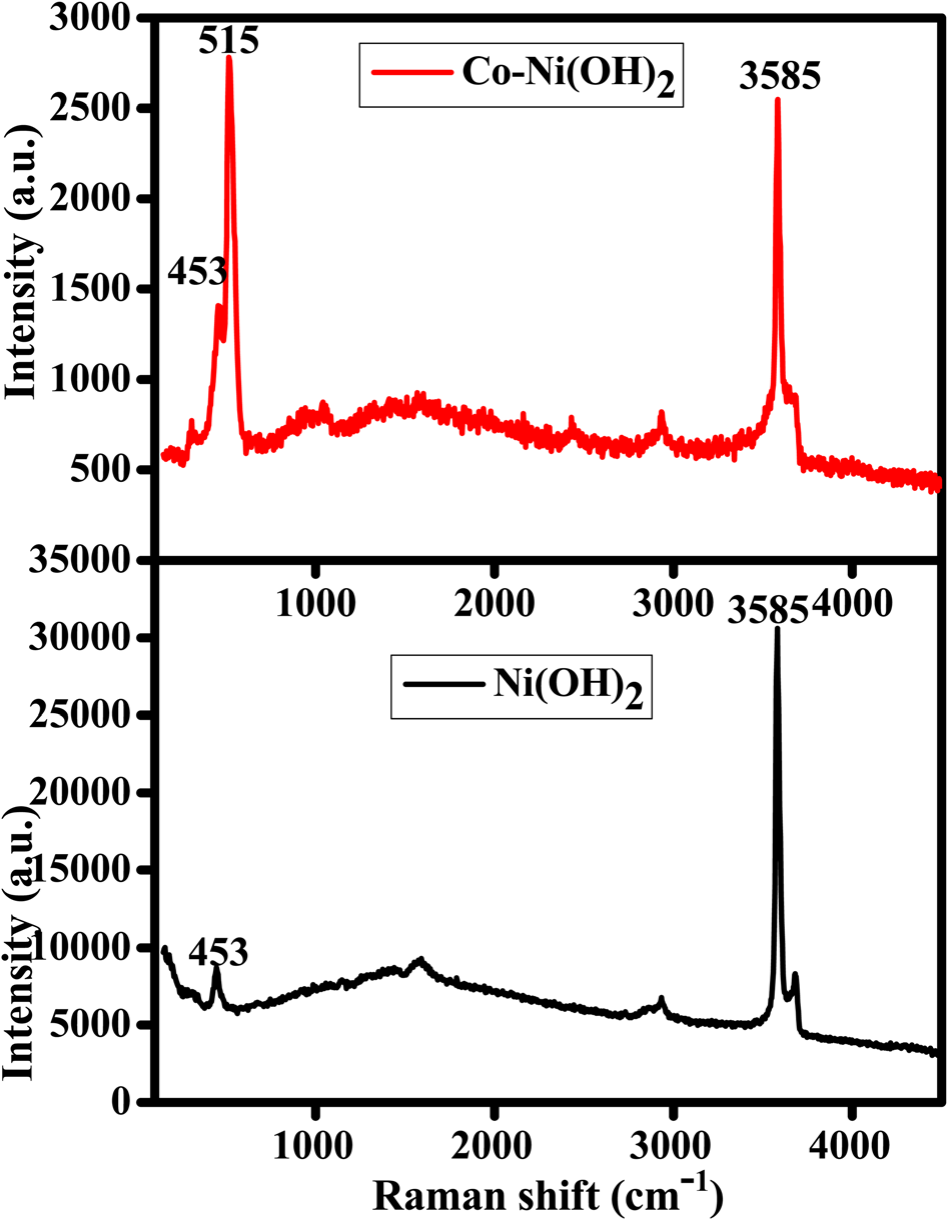
Raman spectra of Co-Ni(OH)_2_ and Ni(OH)_2_.

An independent expert has viewed the corrected Fig. 3 and the associated raw data and confirmed that it is consistent with the discussions and conclusions presented.

The Royal Society of Chemistry apologises for these errors and any consequent inconvenience to authors and readers.

